# A platform supporting generation and isolation of random transposon mutants in *Chlamydia trachomatis*

**DOI:** 10.1128/jb.00500-24

**Published:** 2025-02-14

**Authors:** Caroline Hawk, Nur Hamdzah, Zoe Dimond, Kenneth A. Fields

**Affiliations:** 1 Department of Microbiology, Immunology, & Molecular Genetics, University of Kentucky College of Medicine 214561 , Lexington, Kentucky, USA; 2 Host-Parasite Interactions Section, Laboratory of Bacteriology, Rocky Mountain Laboratories, NIAID/NIH 92615 , Hamilton, Montana, USA; University of Illinois Chicago, Chicago, Illinois, USA

**Keywords:** genetics, *Chlamydia*, pathogenesis, mutagenesis

## Abstract

*Chlamydia* species represent a paradigm for understanding successful obligate intracellular parasitism. Despite limited genetic malleability, development of genetic tools has facilitated the elucidation of molecular mechanisms governing infectivity. Random mutagenesis approaches provide one of the most powerful strategies available to accomplish untargeted elucidation of gene function. Unfortunately, initial progress in transposon-mediated mutagenesis of *Chlamydia* has been challenging. To increase efficiency, we developed a plasmid-based system that couples conditional plasmid maintenance with a previously described strategy leveraging inducible expression of the *Himar1*-derived C9 transposase. Our pOri-Tn(Q) construct was maintained in *Chlamydia trachomatis* cultivated with antibiotics but was rapidly cured in the absence of antibiotic selection. pOri-Tn(Q) supported transposition events when transposase expression was induced during infection. Induction was accompanied by loss of the plasmid backbone when penicillin G was used to select for only the transposable element. C9 induction during iterative passaging was used to increase the overall insertion frequency and accumulate an expanded pool of transposon mutants. The approach supported isolation of individual mutant strains from the mixed pool, and whole-genome sequencing confirmed that the recovered strains harbored single insertions.

**IMPORTANCE:**

*Chlamydia trachomatis* is a prevalent human pathogen exerting a tremendous negative impact on human health. A complete understanding of how these bacteria create and maintain an intracellular niche and avoid/subvert host defense mechanisms to cause disease is lacking. The utility of transposon-mediated, random mutagenesis in supporting forward genetic studies is well established in a multitude of genetically tractable systems. This study reports the development of a plasmid-based system capable of generating mutant pools and supporting subsequent isolation of individual transposon mutants. This step is an important advance in providing a mechanism capable of supporting downstream studies interrogating chlamydial biology.

## INTRODUCTION

Members of the genus *Chlamydia* infect a range of eukaryotic hosts, but *Chlamydia trachomatis* (*Ctr*) represents a prevalent and strict human pathogen (1). *Ctr* serovars display tissue tropism in that ocular infections with serovars A–C can lead to blinding trachoma while serovar D–K infections of the urogenital tract mediate the most common bacteria-mediated sexually transmitted disease in the United States (2). Lymphogranuloma venereum (LGV) serovars L1–L3 also infect the genital tract but can traverse the mucosa to reach regional lymph nodes (3). *Ctr* infections can have serious negative impacts on female reproductive health such as ectopic pregnancy and infertility (4). Re-infections are common, and without an effective vaccination to lower the frequency of infection, *Ctr* poses an ever-increasing threat to public health (5).

*Ctr* is a gram-negative, obligate intracellular bacterium that preferentially replicates in epithelial cells of respective mucosae. All *Chlamydia* spp. possess a bi-phasic developmental cycle (6) that is initiated by invasion of a host cell by the metabolically dormant elementary body (EB). Once intracellular, EBs differentiate into the vegetative, but non-infectious reticulate body (RB) form that actively replicates until an unknown signal triggers asynchronous transition back into EBs. Development occurs entirely within a membrane-bound vacuole termed the inclusion and is concluded by lytic or non-lytic exit from the infected cell (7). Pathology indicative of *Chlamydia*-mediated disease is driven primarily by non-productive immune responses (8), and the infected epithelial cell is capable of initiating and driving immunopathology (9). This has placed a high priority on understanding the cellular microbiology of chlamydial infection. Progress in elucidating mechanistic details governing infectivity has been greatly facilitative by recent advances in genetic manipulation of chlamydiae (10).

Historically, the obligate intracellular nature and biphasic developmental cycle contributed to the low genetic tractability of *Ctr* (11). Recent progress in the field has established a reproducible means for transformation, selection, and shuttle vector capability (10). The latter was made possible using the endogenous plasmid found in multiple *Chlamydia* species (12) as a backbone enabling stable maintenance of recombinant plasmids (13). Engineered shuttle vectors have been used for ectopic expression of chlamydial genes and to enable reverse genetic approaches using targeted gene inactivation (10). Mutagenesis approaches include the use of Group II introns (TargeTron) to inactivate targeted genes via site-specific insertion (14). Complete gene deletions have been created using conditionally replicating plasmids. Coupled with fluorescence markers and *Chlamydia*-specific targeting sequences, these plasmids enable fluorescence-reported allelic exchange mutagenesis (FRAEM) for deletion of specific DNA (15, 16). CRISPRi has been used for conditional knock-down of essential protein expression by conditional expression of dCas9 and sequence-specific guide RNAs (17–19). Despite these advances, gaps remain in our knowledge of chlamydial infection biology. Sole reliance on targeted approaches limits the ability to fully elucidate mechanistic details of biology supporting chlamydial infection.

Transposon (Tn)-mediated mutagenesis creates easily identifiable and traceable mutations, rendering it a powerful tool to support both forward and reverse genetic approaches. Tn-mediated, random mutagenesis coupled with NGS has a proven track record in supporting high-throughput, discovery-based studies (20). Indeed, progress has been made in *Chlamydia* using the *Himar1*-derived transposase (10). Limited transposition in *Ctr* and *Chlamydia muridarum* (*Cm*) was achieved using repeated transformation of bacteria with transposase systems encoded on non-replicating donor plasmids (21, 22). Most recently, low transformation efficiency was overcome using a lower activity C9 transposase allele expressed from a stably maintained shuttle vector. Inducible transposon expression was mediated by a *tet* promoter and synthetic riboswitch to provide transcriptional and translation control of C9, respectively (23). Unfortunately, stable maintenance of the donor plasmid precluded isolation of individual strains and raised the possibility of runaway transposition depleting mutant pools (23).

The chlamydial plasmid encodes eight open-reading frames with a requirement of CDS3/Pgp1, CDS4/Pgp2, and CDS8/Pgp6 for plasmid maintenance (24, 25). CDS2/Pgp8 (26) and the ca. 200 bp origin of replication (ori) (15) determine species specificity. Conditional maintenance of recombinant plasmids has been achieved by conditional expression of CDS8/Pgp6 (16) or by excluding all endogenous plasmid sequences except the iteron (15). In this study, we aimed to engineer a conditionally replicating plasmid platform based on the inducible C9 system developed by O’Neill et al. (23). We leveraged the pL2 interon to achieve selectable plasmid maintenance. We additionally show that this plasmid can generate pools of random mutants that can be selected using antibiotic resistance encoded on the transposable element. Finally, we demonstrate a proof-of-principle approach to isolate individual mutants from the mixed pool. This system promises the capacity for larger scale work to accomplish both random and arrayed libraries of *C. trachomatis* insertion mutants.

## RESULTS

**Fig 1:**
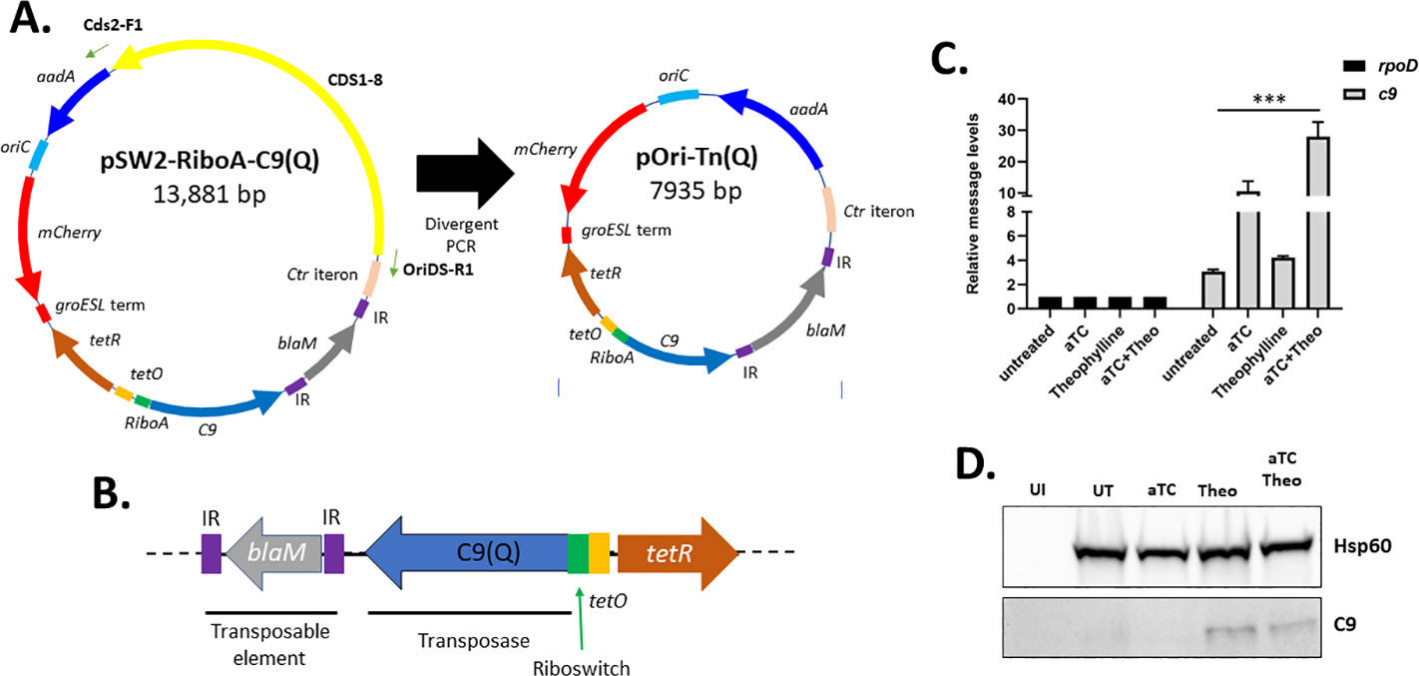
Construction of pOri-Tn(Q) and confirmation of conditional C9 expression. (**A**) Schematic of pSW2-RiboA-C9 (Q) and pOri-Tn(Q) indicating positions of the transposable element containing *blaM* flanked by IR, the low-activity C9(R131Q) allele, regulatory elements (*tetO* and RiboA), mCherry, Spec^r^ (*aadA*), and the *Ctr* (iteron) and *Escherichia coli* (*OriC*) origins of replication. CDS1–8 (yellow) was deleted from pSW2-RiboA-C9(Q) using divergent PCR primers Cds2-F1 and OriDS-R1 to yield pOri-Tn(Q). (**B**) Arrangement of the transposable element, transposase, and regulatory elements tetO and Riboswitch remains conserved. (**C**) Quantitative reverse trasncribe polymerase chain reaction (qRT-PCR) of *c9* message in cultures uninduced or induced for 24 hours with 50 ng/mL aTc and/or 2 mM Theo. Levels of chlamydial *rpoD* were analyzed as a normalization control. Message levels for *c9* showed significant changes (***P < 0.0001 one-way analysis of variance {ANOVA}). (**D**) C9 protein levels in cultures induced for 24 hours. Immunoblots were probed with anti-C9 or chlamydial Hsp60 as a loading control. Data are representative of three experiments.

Previous advances in developing a transposon-mediated, forward genetic approach in *Chlamydia trachomatis* L2 (*Ctr* L2) used regulated transposase expression but did not support purification of isolates from a complex mutant pool (23). Stable maintenance of the transposase-encoding plasmid was likely one confounding factor. We reengineered pSW2-RiboA-C9(Q) using insertion-deletion PCR to remove the pL2 coding sequences (CDS1–8) while retaining the *Ctr* iteron (Fig. 1A). The resulting plasmid maintained the Q allele of C9 transposase (27). The inducible control elements including a Tet promoter (controlling transcription) and a theophylline (Theo)-dependent riboswitch RiboA (controlling translation) were also retained from the pSW2-RiboA-C9(Q) construct (Fig. 1B). The transposable element contains the antibiotic selection cassette *blaM* (PenG) flanked by inverted repeats (IR) whereas *aadA* (spectinomycin [Spec]) is encoded on the plasmid backbone. The new construct, designated pOri-Tn(Q), was transformed into *Ctr* L2 using Spec selection.

We confirmed that reengineering the plasmid did not impact inducible regulation. Transcriptional regulation via the Tet-inducible promoter was evaluated using qRT-PCR to quantify mRNA levels of *c9* relative to *rpoD* (Fig. 1C). HeLa cultures were infected with *Ctr* expressing pOri-Tn(Q) and treated 12 hours post infection with either anhydrotetracycline (aTc), Theo, or both. Untreated cultures were included as a control. As expected, *c9*-specific message was induced in the presence of aTc but not when Theo was present alone. We noted that the *c9* message was still detectible in untreated cultures and conclude that the *tet* promoter is not completely silent in the absence of exogenous aTc. We confirmed riboswitch-mediated translational regulation by probing for the presence of the C9 protein in immunoblots (Fig. 1D). Uninfected HeLa cells were used as an antibody specificity control. Consistent with pSW2-RiboA-C9Q (23), protein was only detected in the presence of Theo. These results show that the inducible control elements remained functional in the new construct of pOri-Tn(Q).

**Fig 2:**
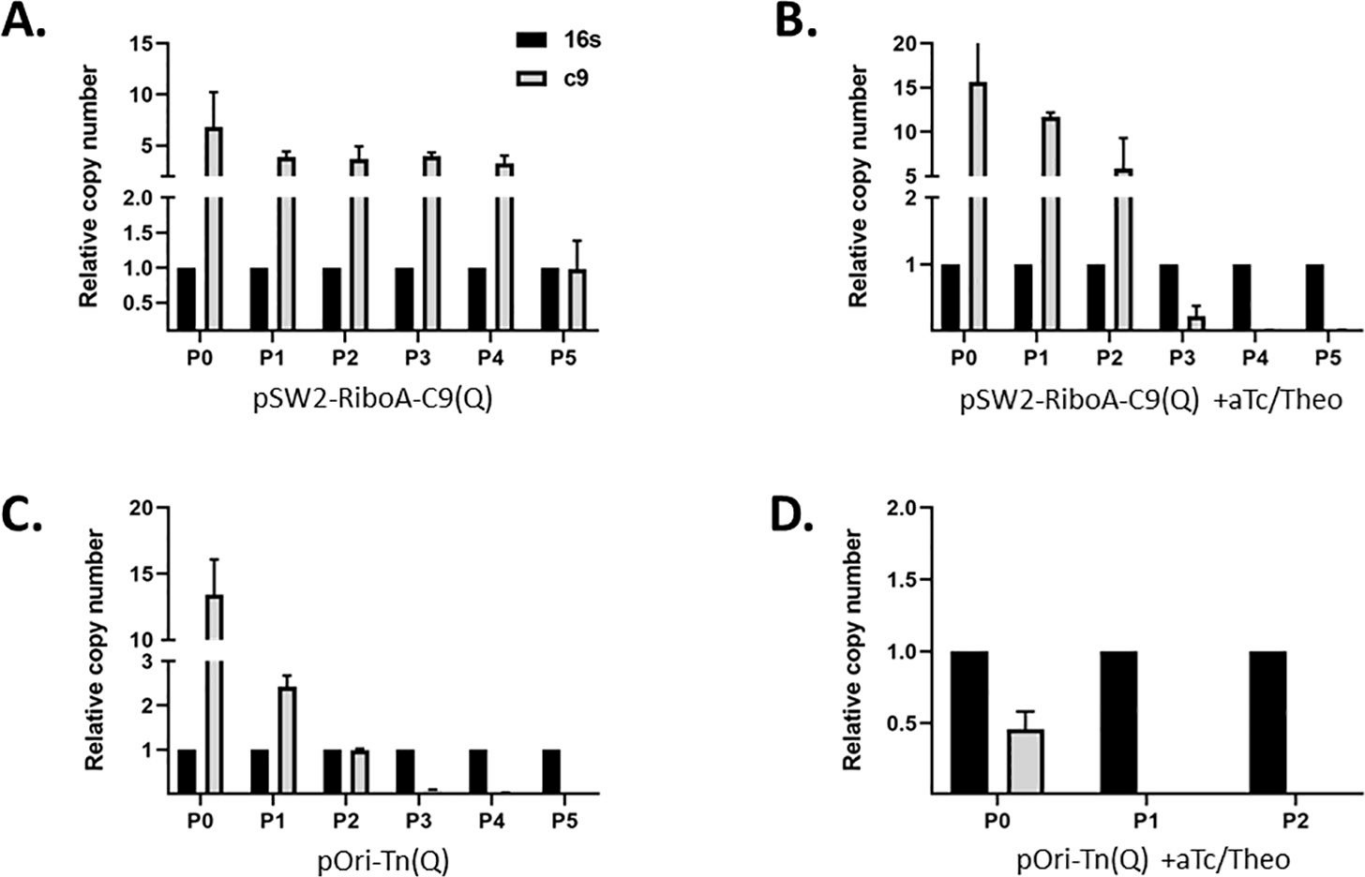
Assessment of plasmid curing via qPCR analysis of relative c9 levels. *Ctr* L2 expressing either pSW2-RiboA-C9(Q) (**A and B**) or pOri-Tn(Q) (**C and D**) was serially passaged (P0–P5) every 48 hours in the absence of Spec selection. DNA was harvested at each passage and C9 levels, normalized to chromosomal 16S, and determined by qPCR. *Ctr* L2 pSW2-RiboA-C9(Q) cultured in the absence (**A**) or presence (**B**) of aTc and Theo induction. *Ctr* L2 pOri-Tn(Q) cultured in the absence (**C**) or presence (**D**) of aTc and Theo induction. Data are presented as mean (±standard deviation) from triplicate cultures.

Our mutagenesis strategy depends on conditional maintenance of the plasmid backbone encoding C9. Plasmid incompatibility between pOri-Tn(Q) and pL2 should result in pOri-Tn(Q) curing when antibiotic selection is absent. We addressed this parameter by qPCR-based quantification of C9 during serial passage of cultures without antibiotic selection (Fig. 2). This was accomplished by infecting HeLa cells, in the presence of Spec, with *Ctr* L2 expressing either pSW2-RiboA-C9(Q) or pOri-Tn(Q). Cultures were diluted to maintain culture size and serially passaged up to six times (P0–P5) to new monolayers every 48 hours and cultivated in the absence of antibiotic selection. DNA was extracted at each passage, and plasmid abundance was assessed using qPCR to quantify C9 relative to chromosomal 16S. Loss of pSW2-RiboA-C9(Q) was not observed in the absence of selection as the C9 signal was detected in all passages (Fig. 2A). We did, however, observe eventual curing of pSW2-RiboA-C9(Q) in the presence of C9 induction (aTc + Theo). Plasmid levels remained abundant for three passages (P0–P2), but the signal was below detection by passage 4 (Fig. 2B). We next examined pOri-Tn(Q) maintenance. Levels of pOri-Tn(Q) steadily declined in the absence of antibiotics and were below detection by P3 (Fig. 2C). Curing was enhanced in the presence of transposase inducers where C9 was below detection after one passage (Fig. 2D). Collectively, these data confirm rapid curing of pOri-Tn(Q) and raise the possibility of generating strains harboring single insertion mutations via induction of transposition followed by loss of the transposase. Eventual curing of pSW2-RiboA-C9(Q) in the presence of aTc and Theo is consistent with prolonged C9 exposure being detrimental.

**Fig 3:**
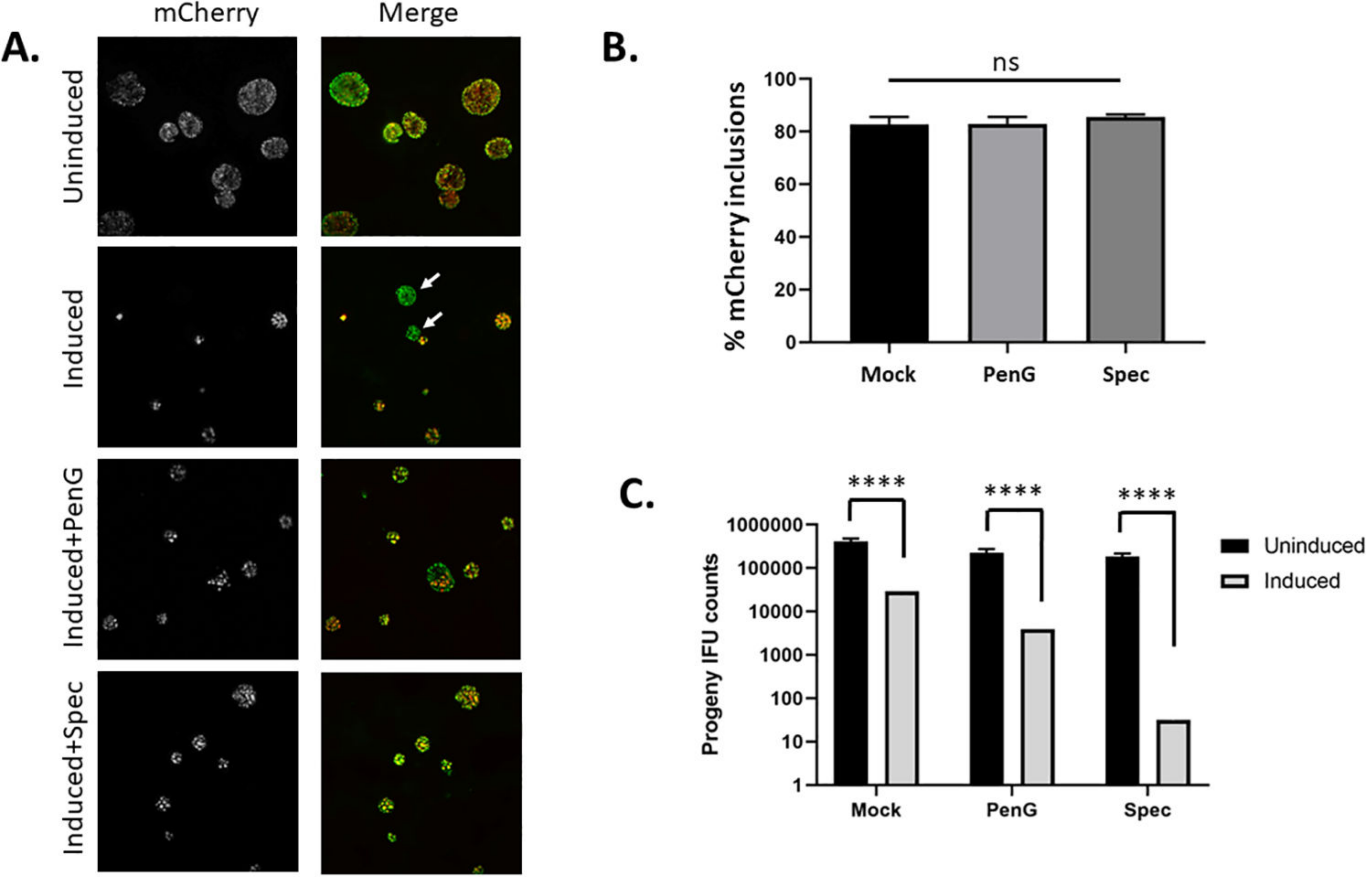
Impact of antibiotic selection during C9 induction. HeLa cells were infected with *Ctr* L2 expressing pOri-Tn(Q) and cultured for 24 hours in the absence (uninduced) or presence (induced) of aTc and Theo. Respective cultures were left untreated (Mock) or supplemented with either PenG or Spec. Cultures were fixed and stained for chlamydial inclusions (**A and B**) or harvested for passaging onto fresh HeLa monolayers, without any treatment, for enumeration of progeny (**C**). (**A**) All inclusions were visualized by staining with Hsp60 antibodies followed by Alexa-488-conjugated secondary antibodies. (**A**) Fluorescence images showing inclusions retaining mCherry signal from pOri-Tn(Q) (red) and total inclusions (green). Arrows indicate green-only inclusions lacking pOri-Tn(Q). Bar = 10 µm. (**B**) Quantitation of mCherry (pOri-Tn(Q)+)-positive inclusions in primary cultures indicated no significant difference. All data are shown as means of triplicate samples (±standard deviation). Statistical significance was tested using one-way ANOVA with Tukey multiple comparisons (**B**) or multiple *t*-tests using Holm-Sidak correction (**C**) (ns = not significant; ****, adjusted *P* value < 0.0001).

We next sought to establish selective conditions for optimal generation of transposon mutants that balanced transposition, the possible toxicity of C9, and plasmid curing. In a pilot experiment, we infected HeLa cells and induced aTc and Theo in the absence of antibiotics. We failed to detect any transposon insertions via Illumina DNA sequencing of genomic DNA (gDNA) harvested 24 hours after induction, indicating the need for selective pressure to maintain pOri-Tn(Q) long enough to ensure transposition. There are two options for pOri-Tn(Q), which encodes *aadA* (Spec) on the plasmid backbone and *blaM* (PenG) within the transposable element. To evaluate the impact of these antibiotics, we leveraged mCherry encoded on the plasmid backbone as a visual indicator for the presence of pOri-Tn(Q). Infected HeLa cells were either untreated or induced (aTc + Theo) with the addition of PenG or Spec at 12 hours after infection. Cultures were paraformaldehyde fixed 24 hours after induction and stained with HSP60 conjugated to Alexa-488 to visualize total inclusions. Under induction, we observed reduced inclusion size and abnormal inclusion morphology regardless of antibiotics (Fig. 3A). We also routinely detected mCherry-deficient inclusions in both the absence and presence of antibiotics. The pOri-Tn(Q)-positive inclusions were quantified in cultures induced in the presence of PenG or Spec by enumeration of mCherry-positive inclusions (Fig. 3B). Infected HeLa cells cultured with induction, but not antibiotic, served as a control. Loss of mCherry in these primary induced cultures was not impacted by inclusion of antibiotics. mCherry-deficient inclusions were detected in 17.2% (±2.84%) of cultures without antibiotics and in 17.1% (±2.6%) or 14.4% (±0.96%) in cultures containing PenG or Spec, respectively.

We quantified progeny EBs to gauge the overall impact of induction conditions on chlamydial fitness. *Ctr* pOri-Tn(Q)-infected HeLa cells were either uninduced or induced for 24 hours in the absence or presence of individual antibiotics. Cultures were harvested after 24-hour induction periods and passaged to new monolayers (without antibiotics) to enumerate progeny (Fig. 3C). Uninduced cultures cultivated under the three treatment conditions displayed similar progeny numbers. Progeny counts were significantly reduced, however, in the presence of induction. The impact was most severe (10^−4^ reduction) in the presence of Spec but also manifested to a lesser degree in the absence of antibiotics (ca. 10^−1^ reduction) or presence of PenG (ca. 10^−2^ reduction). Reduced progeny was not due to aTc and Theo since addition of inducers to wild-type (WT) *Ctr* L2 did not impact fitness. Progeny from infected cultures treated with aTc/Theo for 24 hours (4.88 × 10^8^ [±9.6 × 10^7^]) did not significantly differ from parallel mock-treated cultures (2.65 × 10^8^ [±1.43 × 10^8^]). These data indicate that the presence of antibiotic did not affect plasmid maintenance in primary cultures during the induction process. Selective pressure, however, resulted in decreased recovery of viable bacteria, especially in the case of co-cultivation with Spec.

**Fig 4:**
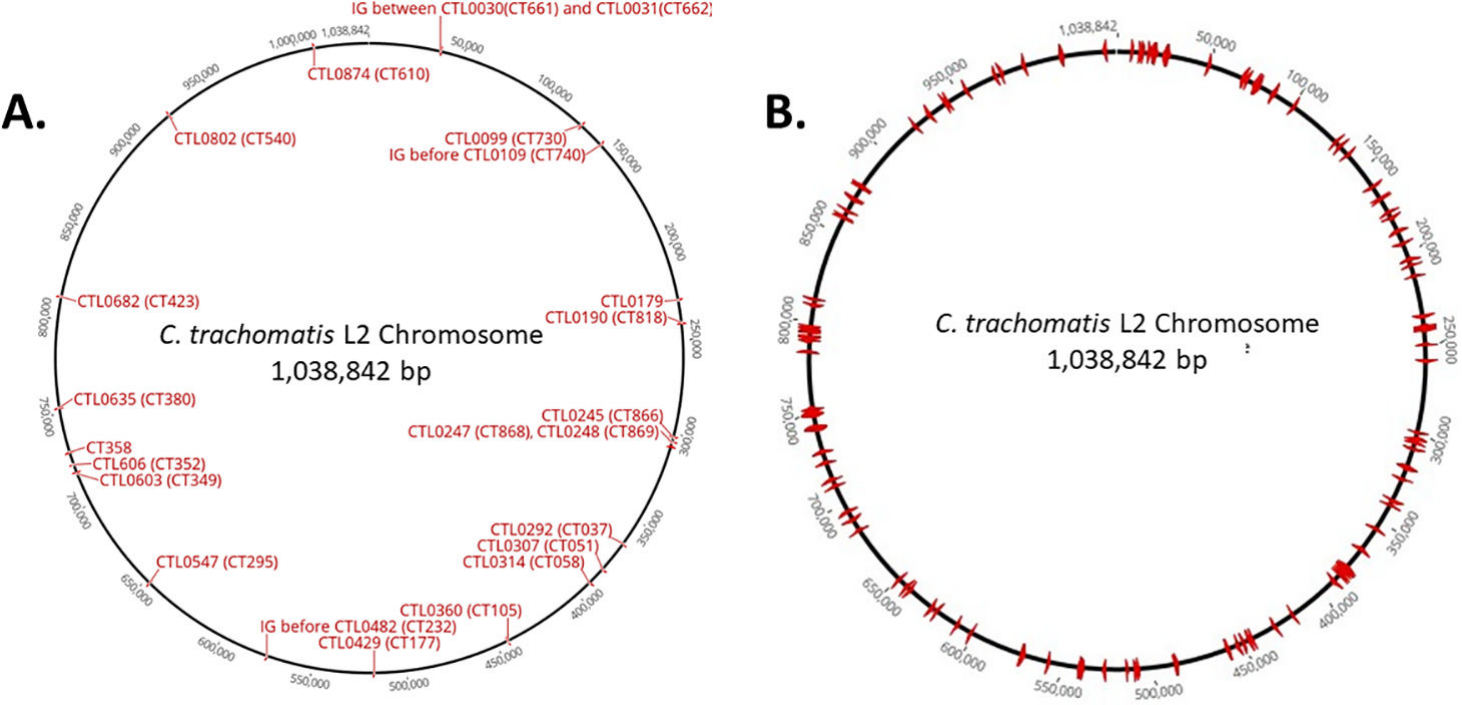
Schematic indication of transposon insertion sites. HeLa cell cultures were *Ctr* L2 pOri-Tn(Q) and induced with aTc + Theo in the presence of PenG either during primary culture (**A**) or during iterative passaging (**B**). Harvested gDNAs were subjected to Illumina WGS at equivalent depths. Respective transposon insertions were mapped and are shown schematically. Corresponding gene loci (red) are shown for primary culture insertions (**A**) whereas chromosomal positioning is indicated for the more numerous inserts derived from passaging (**B**). Chromosome positions are presented numerically and annotated in increments of 50,000 bp.

We reasoned that selection for the transposable element with PenG during induction of C9(Q) expression could allow transposition while simultaneously enabling loss of the pOri-Tn(Q) backbone. HeLa cells (ca. 5 × 10^6^) were infected with *Ctr* pOri-Tn(Q) at an MOI of 1 and cultivated for 18 hours in the presence of PenG. Media were then supplemented with aTc and Theo, and the cultures were incubated for an additional 24 hours. Cultures were harvested for direct gDNA extraction and subsequent Illumina whole genome sequencing (WGS). The resulting sequencing reads were mapped to the *C. trachomatis* L2/434/Bu reference genome using Geneious software. A total of 22 insertions were identified with three mapping to intergenic regions on the chromosome (Fig. 4A). The remaining insertions mapped to coding sequences distributed throughout the chromosome. These data confirmed that pOri-Tn(Q) possessed mutagenic capability.

Given the relatively low number of insertions in primary cultures, we next tested whether PenG-mediated selection of the transposable element coupled with serial induction would increase the overall number of transposition events. HeLa cultures were infected at an MOI of 1 with ca. 3 × 10^5^ infectious forming units (IFU) and supplemented with Theo, aTC, and PenG at 18 hours post infection. Cultures were harvested and expanded twice by passaging two additional times under C9(Q) induction with PenG antibiotic selection. Cultures were split into four aliquots of 1.0 mL of SPG and stored at −80°C. gDNA was extracted from one aliquot containing ca. 2 × 10^9^ IFU and subjected to WGS. A total of 143 unique insertions were detected distributed throughout the chlamydial genome (Fig. 4B). Individual insertions were compiled with the gene identity, putative function, protein truncation, and corresponding genomic gene designation (Table S1). Twenty-eight insertions were detected within intergenic regions whereas the remaining 111 appeared within chromosomal CDS. Three insertions were detected within genes on the endogenous plasmid. The pool harbored multiple insertions for some genes including six unique insertions in *pmpB*; four in *dusB*; three in *tmeA*, *tyrP*, *ctl0447*, *ctl0625*, and *pmpA*; and two each in *recC*, *ctl0019*, *parB*, *uvrC*, *hemW*, sucA, *sfhB*, *hflX*, *phnP*, *ctl0738*, *ctl0743*, and *ctl0874*. A variety of gene truncations were noted, ranging from insertion after codon 2 for the hypothetical *ctl0523* to the final codon in *secA*, *rsmA*, *pmpA*, and *xerD*. 34.5% of insertions truncated at least half of the respective coding sequence. In aggregate, these data indicate that iterative cultivation under inducing conditions, while selecting for the transposable element, can be a productive means to generate insertion mutants. We next sought to address whether the platform could support clonal isolation of mutant strains that would then enable specific validation of insertions.

We employed limiting dilution of the mutant pool in 384-well plates to test whether individual stains could be recovered. Material from the twice-passaged induction was diluted and used to infect one 384-well plate with predicted 200 EBs. Wells containing non-fluorescent, PenG-resistant inclusions were harvested at 7–10 days and expanded on fresh HeLa monolayers in the presence of PenG. A total of 50 wells were harvested. Aliquots of expanded strains were mixed equally (based on IFU) in groups of five to six for gDNA extraction and WGS of small pools. Sequencing identified 41 unique insertions (Table 1). Identical insertions in *ctl0462* and *ctl0895* were detected within two pools indicating duplicate isolation. Nineteen of the insertions were new and not detected in the original mutagenesis sequencing pool (Table S1). Insertions were represented in intergenic regions (11), genes with predicted (21) or unknown function (11), and the endogenous plasmid (1). Use of transcriptional start sites identified by the *C. trachomatis* transcriptome analysis performed by Albrecht et al. (28) indicated that 15 of the insertions likely occur within polycistronic operons (Table 1).

**TABLE 1:** Insertions identified from sequencing pools

Direction^*a*^	Gene^*b*^	Putative function	Protein change^*c*^	CTL^*d*^	CT^*e*^	Polycistronic^*f*^
Forward	*trmB*	tRNA (guanosine(46)-N7)-methyltransferase TrmB	168/225aa	CTL0201	CT_829	Y, 3/3
Reverse	*hemL*	Glutamate-1-semialdehyde 2,1-aminomutase	183/423aa	CTL0462	CT_210	N
Reverse	*pmpA*	Pmp family polymorphic membrane protein autotransporter adhesin	701/976aa	CTL0669	CT_412	Y, 1/3
Forward	*yceA*	Rhodanese-related sulfurtransferase CDS	255/328aa	CTL0891	CT_627	N
Reverse	*yabC1*	SAM-dependent methyltransferase	95/238aa	CTL0304	CT_048	Y, 1/2
Reverse	*cppA*	Serine/threonine-protein phosphatase	28/249aa	CTL0511	CT_259	Y, 1/2
Reverse	*inaC*	Inclusion membrane protein	64/265aa	CTL0184	CT_813	Y, 4/4
Reverse	*ttcA*	tRNA 2-thiocytidine biosynthesis TtcA	76/255aa	CTL0469	CT_217	N
Reverse	*hflX*	GTPase HflX	390/448aa	CTL0634	CT_379	Y, 2/3
Reverse	*maf*	Septum formation Maf-like protein	169/197aa	CTL0603	CT_349	Y, 2/4
Forward	*tyrP*	Aromatic amino acid transport family protein	88/398aa	CTL0190	CT_818	N
Reverse	*yycJ*	MBL fold metallo-hydrolase	79/263aa	CTL0107	CT_738	Y, 2/3
Reverse	*dub2*	Deubiquitinase	126/340aa	CTL0246	CT_867	Y, 1/4
Forward	*mot1*	DEAD/DEAH box helicase	1187/1200aa	CTL0818	CT_555	Y, 1/3
Reverse	*lda2*	Hypothetical protein; lda2	152/520aa	CTL0419	CT_163	Y, 5/5
Reverse	bioY	Biotin transporter BioY	174/197aa	CTL0613	CT_359	N
Reverse	tlyC	Hemolysin family protein	369/370aa	CTL0682	CT_423	N
Reverse	nfo	Deoxyribonuclease IV	278/289aa	CTL0889	CT_625	N
Reverse	*incM*	Inclusion membrane protein	561/565aa	CTL0540	CT_288	N
Reverse	*dre1*	Inclusion membrane protein	46/242aa	CTL0444	CT_192	N
Intergenic						
Reverse	Intergenic	N/A	N/A	IG before CTL0260	IG before CT_ 005	IG before 1/2
Forward	Intergenic	N/A	N/A	IG before CTL0663	IG before CT_406	IG before 1/2
Reverse	Intergenic	N/A	N/A	IG before CTL0455	IG before CT_203	N
Forward	Intergenic	N/A	N/A	IG after CTL0091	IG after CT_772	IG after 1/3
Forward	Intergenic	N/A	N/A	IG before CTL0250	IG before CT_871	IG before 1/2
Reverse	Intergenic	N/A	N/A	IG after CTL0269	IG after CT_ 014	IG after 2/2
Reverse	Intergenic	N/A	N/A	IG before CTL0662	IG before CT_405	IG before 1/4
Forward	Intergenic	N/A	N/A	IG before CTL0007, IG after CTL0006	IG before CT_639	N
Reverse	Intergenic	N/A	N/A	IG before CTL0814	IG before CT_552	IG before 4/5
Forward	Intergenic	N/A	N/A	IG after CTL0615	IG after CT_361	N
Reverse	Intergenic	N/A	N/A	IG before CTL0852	IG before CT_589	IG before 2/2
Hypothetical proteins						
Reverse	Hypothetical protein CDS	Hypothetical protein	59/92aa	N/A	CT_037	Y, 2/3
Reverse	Hypothetical protein CDS	Hypothetical protein	129/243aa	CTL0271	CT_016	N
Reverse	Hypothetical protein CDS	Hypothetical protein	229/269aa	CTL0535	CT_283	N
Forward	Hypothetical protein CDS	UvrB/UvrC motif-containing protein	12/174aa	CTL0045	CT_676	Y, 1/2
Reverse	Hypothetical protein CDS	Hypothetical protein	257/823aa	CTL0113	CT_744	N
Reverse	Hypothetical protein CDS	DUF1347 family protein; putative exported lipoprotein	596/609aa	CTL0019	CT_651	N
Reverse	Hypothetical protein CDS	Lipoprotein	221/222aa	CTL0103	CT_734	Y, 1/3
Reverse	Hypothetical protein CDS	DUF3604 domain-containing protein	622/622aa	CTL0684	CT_425	Y, 1/4
Forward	Hypothetical protein CDS	Hypothetical protein	85/85aa	CTL0895	CT_631	N
Plasmid						
Reverse	pgp7	Integrase pGP7-D	160/305aa		pCT07	

^*a*^ Direction indicates orientation of the transposable to the sense (forward) or anti-sense (reverse) strand of the chromosome.

^*b*^ Gene designations are listed as indicated in annotated chlamydial genome (PMID 9784136). Genes encoding proteins of unknown function are listed “Hypothetical protein CDS,” and insertions occurring outside of an apparent CDS are designated “intergenic.”

^*c*^ Protein change is indicated by the last residue before transposon direction/total number of residues in the CDS. Positions of intergenic insertions (IG) are indicated relative to the adjacent coding sequence.

^*d*^ Gene designations are derived from the *C. trachomatis* L2 chromosome annotation (PMID 18032721). Positions of IG are indicated relative to the adjacent coding sequence.

^*e*^ Corresponding gene designation form the *C. trachomatis* serovar D genome is indicated based on PMID 9784136.

^*f*^ “Y” and “N” are used to designate whether transcriptome analysis (PMID 19923228) indicated the corresponding locus occurs within a polycistronic message. The position of the gene harboring the insertion is indicated along with the total number of coding sequences in the respective message.

**Fig 5:**
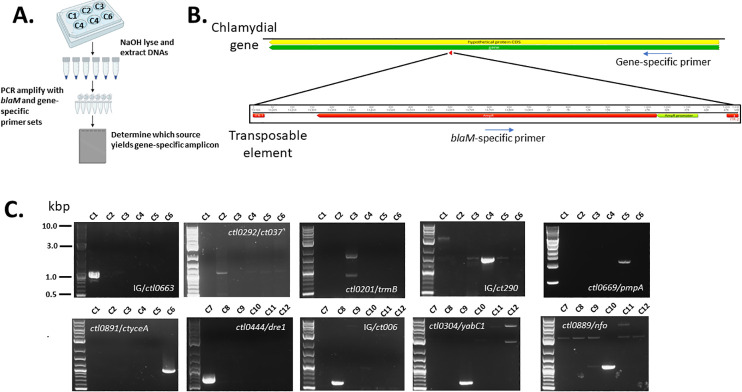
Identification of clonal strains. (**A**) Schematic representation of screening process where DNA is extracted from individual strains cultivated for 24 hours in six-well plates. PCR allows identification of which clone corresponds to a given insertion. (**B**) Generalized scheme of PCR primer design. A gene-specific primer is located within the disrupted coding sequence and used in conjunction with a transposon-specific *blaM* primer. (**C**) Respective PCR reactions were resolved and visualized in 1% agarose gels. Products from 10 isolates derived from WGS groups 1–6 and 1–12 were included on separate gels. DNA size standards were included, and 0.5, 1.0, 3.0, and 10.0 kbp bands are indicated.

As proof-of-principle, we chose to specifically identify insertions occurring in 10 clonal strains represented in the first two sequencing pools. These included insertions within three intergenic regions, six genes with known/predicted function (*trmB*, *pmpA*, *yceA*, *dre1*, *yabC1*, and *nfo*), and one gene (*ctl0292*/*ct037*) of unknown function. Screening was accomplished by extracting crude DNA from all clonal isolates, which were derived from sequencing groups 1–6 and 7–12 (Fig. 5A). Culture material was derived from 24-hour cultures of Hela cells infected with ca. 10^6^ IFU of respective strains. DNA templates were then probed with gene-specific primers paired with a primer annealing within *blaM* of the transposon (Fig. 5B). Visualization of PCR products on agarose gels identified the identity of all 10 clonal isolates. Amplicons appeared only in single samples indicating successful clonal isolation of strains. gDNA extracted from each strain was subjected to WGS to confirm mutations and test whether each carried only a single insertion. Sequencing confirmed insertion loci and that all isolates harbored only the single transposon insertion (Table 2).

**TABLE 2:** Summary of whole genome sequencing of isolates

Isolate	Chromosome coverage^*a*^	Chromosome location^*b*^	Truncation (aa)^*c*^	# Insertions detected	CT	CTL
C1	58X	783,463	Intergenic	1	IG upstream of *ct_406*	IG upstream of *ctl0663*
C2	93 X	364,665	56/92aa	1	*ct_037*	*ctl0292*
C3	143X	259,134	167/225aa	1	*ct_829*	*ctl0201*
C4	107X	643,243	Intergenic	1	IG between *ct_289* & *ct_290*	IG between *ctl0541* & *ctl0542*
C5	29X	791,964	701/976aa	1	*ct_412*	*ctl0669*
C6	221X	1,033,762	254/328aa	1	*ct_627*	*ctl0891*
C7	86 X	534,878	45/242aa	1	*ct_192*	*ctl0444*
C8	198X	328,520	Intergenic	1	IG between *ct_005* & *ct_006*	IG between *ctl0260* & *ctl0261*
C9	168X	375,830	96/238aa	1	*ct_048*	*ctl0304*
C10	131X	1,031,859	279/289aa	1	*ct_625*	*ctl0889*

^*a*^ Values indicate genome coverage of sequencing runs.

^*b*^ Transposon insertion locus is indicated based on the LGV 434/Bu sequence NCBI accession no. NC_021052.1.

^*c*^ Protein change is indicated by the last residue before transposon direction/total number of residues in the CDS.

## DISCUSSION

The utility of transposon-mediated mutagenesis in elucidating the genetic basis of pathogenesis is now well established, especially with the advent of cost-effective, high-throughput DNA sequencing technologies (20, 29). Factors such as low transformation efficiency, complex developmental cycles, and a requirement for cultivation under selective conditions have limited full application of transposons in obligate intracellular bacteria. Despite these challenges, transposon systems of variable efficiency have been established for *Anaplasma* (30), *Coxiella* (31), *Ehrlichia* (32), and *Rickettsia* (33–35) where they have been exploited to understand infection biology (10). The advent of reproducible transformation in *Chlamydia* (13) has supported several reverse genetic approaches such as targeted group II interon insertion (14), fluorescence-reported allelic exchange mutagenesis (16), and CRISPRi (17). Development of transposon-based mutagenesis, however, has lagged in development. Herein, we re-engineered pSW2-RiboA-C9Q (23) and optimized induction parameters to yield a platform capable of supporting comparative levels of transposition and direct isolation of clonal mutants.

Initial studies of transposon mutagenesis in *Chlamydia* employed transient expression of the C9 transposase from non-replicating shuttle vectors (21, 22) and demonstrated the efficacy of the *himar1*-based mariner transposase in *Chlamydia*. This approach also resulted in the identification of a potential competence gene in *C. trachomatis* (22) and initial characterization of several polymorphic membrane proteins in *C. muridarum* (21), underscoring the potential of transposon mutagenesis in *Chlamydia*. The low transformation efficiency of *Chlamydia*, however, required repeated rounds of transformations and confounded generation of complex mutant libraries. O’Neill et al. (23) overcame low transformation efficiency by regulated expression of a less active C9 allele (R131Q) from a stably maintained plasmid (23). This elegant system yielded a panel of chromosomal insertions, but isolation of individual mutants was not supported. Low transposition frequency and stable maintenance of the plasmid backbone likely precluded differential enrichment and separation of mutant and wild-type pools.

Recombinant plasmids that express all eight endogenous plasmid ORFs are stably maintained in *Chlamydia* (13), and our data confirm that pSW2-RiboA-C9Q remains present after multiple passages even in the absence of selection. Although excision of the transposable element can eliminate the donor plasmid, repair via homologous recombination can also occur (36, 37). In addition, endogenous plasmids are maintained at 5–10 copies in *Chlamydia* (38) and the copy numbers of recombinant plasmids are typically higher (16). Multiple plasmid copies make “donor suicide” an inefficient mechanism to cure transposase after mutagenesis. Plasmids maintained by the chlamydial iteron are rapidly lost in the absence of selective pressure (15), making this an attractive strategy for transient transposase expression. Alteration of pSW2-RiboA-C9Q required only PCR-based deletion of the eight coding sequences. The resulting plasmid, pOri-Tn(Q), retained the transposon system, selection cassettes, and expression control elements of pSW2-RiboA-C9Q. We confirmed that our changes did not affect C9(Q) expression. The presence of C9 message in uninduced cultures is likely due to incomplete TetR repression, but we did not rule out the possibility of residual tetracycline in culture media since we did not use certified Tet-free fetal bovine serum (FBS). Consistent with previous observations (23), we did not detect high levels of C9 in the combined presence of aTc and Theo. This is likely due to the noted low-level expression permitted by riboswitch A (23, 39). As expected, pOri-Tn(Q) was below detection in chlamydiae that were passaged for more than three iterations. Curing was more rapid when transposase expression was induced with aTc and Theo. In addition to rapid curing, pOri-Tn(Q) features additional advantages. Reliance on only the ca. 122 bp iteron for plasmid maintenance decreases the overall plasmid size. pOri-Tn(Q) is 7.9 kb compared with 13.8 kb for pSW2-RiboA-C9Q. This smaller size resulted in efficient transformation with robust detection of transformants after the first passage. Second, strains maintain the chlamydial plasmid since replication of pOri-based plasmids requires trans-acting factors from the endogenous plasmid (15). This is significant since the endogenous plasmid is a recognized virulence factor (40). Finally, the chlamydial iteron is species specific. The L2-based O*ri* used here can be directly used in other *C. trachomatis* biovars (15) or can be replaced with the O*ri* from other species to enable similar mutagenesis beyond *C. trachomatis*.

Our detection of 22 insertions during primary culture of *Chlamydia* is comparable to the number detected using pSW2-RiboA-C9Q (23) and similar in efficiency to accomplishments in *Anaplasma*, *Ehrlichia*, and *Rickettsia* (10). We chose PenG selection during mutagenesis to expand the number of insertion strains since Spec had a more dramatic detrimental impact on viability. Forced maintenance with Spec could also favor multiple insertions within a single strain. Selection for the transposable element should promote enrichment of mutants while simultaneously allowing loss of the C9 transposase along with the pOri-Tn(Q) backbone. In support of this notion, final WGS sequencing of purified isolates confirmed only single insertions in each strain. The 143 insertions detected by our shotgun WGS of iteratively passaged cultures represent an increase in mutagenesis capability for *Chlamydia* but remain well short of saturating mutagenesis. Indeed, comparison of published mutants (22) with our data set reveals an overlap of only 11 genes, indicating saturation has not been reached in a single pool.

The *Himar1*-derived transposase favors random transposable element insertion at T/A dinucleotide sequences (41). The ca. 1.04 Mb L2 434Bu genome is 41.33% G + C and contains >68,000 potential insertion sites. However, essential genes will not be susceptible to inactivation and *Chlamydia* currently lacks an axenic medium (42) that has propelled saturating mutagenesis in *Coxiella burnetii* (10). The chlamydial genome contains a predicted 889 chromosomal and eight plasmid (pL2) coding sequences. Of the 184 total mutations generated during our study, only 15 (8%) occurred within intergenic regions. Some genes are encoded within operons (28), and CDSs include 45 non-protein tRNA and rRNA genes and 15 putative pseudogenes (43). The core genome common to all *Chlamydiae* is comprised of ~560 genes (44, 45) that are hypothesized to represent absolutely essential gene content. This would leave 329 situationally essential genes susceptible to inactivation. Our mutant pool obviously contains more than the 143 insertions originally detected (Table 1) since 19 of the clonal transposon mutants obtained by our isolation approach were not detected in the original sequencing panel. Technical approaches to increase sensitivity of insert detection will therefore be desirable in the future to enable efficacious forward genetic applications. Treatments to subtract host background DNA, thereby enriching chlamydial sequences, represent one possibility. In addition, the transposable element contains the engineered MmeI restriction sites (23). This addition enables digestion of genomic DNAs after mutagenesis and ligation with primer sequences that allow amplification of insertion sites. Subsequent detection via transposon-directed insertion site sequencing is capable of detecting low-abundance insertions (20).

Conditional loss of pOri-Tn(Q) subsequent to mutagenesis supported isolation of clonal transposon mutants. Insertion identification was facilitated by initial sequencing of combined groups of isolated strains. We used groups of five to six, but the efficient identification we experienced indicates that these group sizes could be increased. Based on insertions identified in groups, PCR of individual isolates was then used as an inexpensive method to rapidly assign identity. This step is important if one needs to recover a specific gene disruption from the overall pool. We leveraged limiting dilution to isolate strains for its comparative convenience but could have missed recovery of highly attenuated mutants. Plaquing is an additional method that could be employed where visually small plaques correspond to less fit strains. This approach has been used successfully to isolate attenuated chemical mutants in *Chlamydia* (46) and transposon mutants in *Rickettsia* (33). Our focus in this study was to enable clonal isolation, and we have not yet addressed fitness of recovered mutants. One of the isolated strains, however, contains a truncation after codon 46 of *ctl0444*. The gene product corresponds to the recently described *dre1* inclusion membrane protein. Compared with the WT, a *dre1* null strain exhibited an eightfold reduction in progeny during cultivation in HeLa cells. These data support the notion that our approach can recover attenuated strains.

The need for additional genetic tools for *Chlamydia* is clear. Two hundred ninety (32%) of proteins in *Ctr* are classified as hypothetical with respect to function (47). In most cases, how gene products with designated putative function contribute to chlamydial infection remains unknown. Leveraging high-throughput approaches to dissect infection biology is therefore an attractive possibility to address gene function. Our platform leads to more efficient generation of mutant pools, opening the door for computational approaches to indicate gene function. For example, sufficient levels of mutagenesis could be attained to support identification of essential genes via Monte Carlo analysis as accomplished in *C. burneti* (48). In parallel, or the absence of full saturation, forward-genetic, gain- or loss-of-function studies could be performed similar to those implemented for complex pools of chemically induced chlamydial mutants (46). As noted above, plaquing of pooled mutants would reveal strains exhibiting decreased fitness. This approach has the added advantage of supporting mutant isolation. Previous studies have also applied random mutant pools to identify chlamydial mutants with reduced susceptibility to IFN-γ (49), deepening our understanding of the ability of *Chlamydia* to evade this host immune response. We acknowledge that isolation of a desired, specific strain from a complex pool could be labor intensive in the absence of overt selective pressure. However, genes implicated in forward genetic screens could easily be inactivated directly for further study via methods such as FRAEM, thereby bypassing the need for clonal isolation. Our ability to isolate individual strains via non-biased limiting dilution raises the possibility of building arrayed strain libraries for reverse genetic studies. For example, effector protein mutant subsets could be assembled and screened as described (22) in cell culture or the murine infection model to identify genes necessary for development and/or pathogenesis. Regardless of application, our findings provide a basis for a more rapid advance in understanding the intricate biology supporting an important human pathogen.

## MATERIALS AND METHODS

### Bacteria and cell culture

*C. trachomatis* serovar L2 (LGV 434) was used as the parent strain in these studies. Chlamydiae were maintained in HeLa 229 epithelial cells (CCL-1.2; ATCC) for routine culture and for genetic manipulations. HeLa were grown in RPMI 1640 medium containing 2 mM L-glutamine (Life Technologies) supplemented with 10% (vol/vol) heat-inactivated FBS (Sigma). All cultures were maintained at 37°C in an environment with 5% CO2 and 95% humidified air. Infections were accomplished by centrifugation of EBs onto cell monolayers at 20°C for 1 hour at 900 × *g*. Where appropriate, cultures were supplemented with 2 µg/mL cycloheximide (Cyclo), 50 ng/mL aTc, and/or 2 mM Theo (Sigma). Selection was accomplished using penicillin G (PenG; Sigma) or Spec (AlphaAesar) at 600 ng/mL or 500 µg/mL, respectively.

### Plasmid construction and strain generation

The transposon-expressing plasmid pOri-Tn(Q) was derived from pSW2-RiboA-C9Q (23) kindly provided by Dr. Ian Clarke (University of Southhampton). CDS were deleted from pSW2-RiboA-C9Q via divergent PCR using custom primers Cds2-F1 (5′-TCACAGGACACGCAACCAC-3′), OriDS-R1 (5′-GAGTTTCAATCGATCCCCTATAAATCCGC-3′), and Q5 DNA polymerase. Amplification products were transformed into *E. coli* and screened for loss of CDS1-8 and retention of the pL2 ori. pOri-Tn(Q) was transformed into *dcm*-/*dam- E. coli* (NEB) and mobilized into *C. trachomatis* L2 using CaCl_2_ chemical transformation as described (15). Regulated expression of C9 transposase was confirmed at the transcriptional and translational levels in cultures harvested at 24 hours post infection. For transcription, one-step qRT-PCR (Bio-Rad) was used to detect c9 message in whole-culture RNA isolated using the Aurum Total RNA extraction kit (Bio-Rad). C9 was amplified using forward (5′-GACCTTCAAACGCTCCAATAAC-3′) and reverse (5′-TGGGATGCGCATGGATATT-3′) primers while *rpoD* was detected as a normalization control using RpoD-s (5′-GCGGTGTTTCCATTGTCGTCATA-3′) and RpoD-as (5′-ATTTCTCTCAGCTCGCGCTTTC-3′). For translational analysis, C9 was detected using specific antibodies kindly provided by Dr. David Lampe (Duquesne University) in immunoblots of whole-culture material.

### Quantitative analysis of plasmid and chlamydiae

Plasmid abundance was assessed during serial passage of cultures under various induction and selection conditions. DNA was extracted from wells at 24 hours during respective passages of *C. trachomatis*-infected monolayers (50). Relative counts of chlamydial *16S* or plasmid-encoded *c9* were determined by quantitative real-time PCR using the Bio-Rad CFX96 Real-Time System (Bio-Rad) and iTaq Universal SYBR Green Supermix (Bio-Rad). Primers for *c9* were those used for qRT-PCR, and 16S-s (5′-CCTGGTAGTCCTTGCCGTAAAC-3′) and 16S-as (5′-TACTCCTCAGGCGGCATACTTA-3′) were used to amplify 16S DNA. Detection of mCherry-derived fluorescence was used as an indicator for the presence of pOri-Tn(Q) in live cultures. Quantitative enumeration of chlamydial IFU was performed via indirect immunofluorescence as described (51).

### Immunodetection and microscopy

For immunoblot analyses, proteins were separated on 4% to 15% SDS-PAGE gels (Bio-Rad) and transferred to 0.45 µm PVDF membranes (Millipore). Primary antibodies were specific for C9 transposase (52) provided by Dr. David Lampe (Duquesne University) or Hsp60 (A57-B9, Santa Cruz). Peroxidase-conjugated secondary antibodies (Sigma) and Amersham ECL Plus (GE Healthcare UK Limited) detection reagent were used to visualize proteins. For indirect immunofluorescence, respective cultures were fixed with paraformaldehyde, permeabilized with 0.1% Triton X100, and probed with primary antibodies specific to MOMP (53) for progeny enumeration or Hsp60 for immunolocalization to detect chlamydiae. Visualization was accomplished using secondary antibodies conjugated to AlexaFluor-594 or -488 (Invitrogen). Cells were examined via epifluorescence microscopy using a Nikon E800 Eclipse with 100× oil immersion objective or via confocal using a Nikon A1R inverted confocal microscope with 63× oil immersion objective. All images were processed equivalently using auto contrast and unsharp-mask filter functions in Adobe Photoshop 6.0 (Adobe Systems).

### Induction of transposition and strain isolation

Induction of the C9 transposase was accomplished as described (23). HeLa cell cultures were infected at an MOI of 1 and incubated for 12 hours with Cyclo and Spec. Media were replaced with RPMI supplemented with PenG, aTc, and Theo. Cultures were incubated an additional 24 hours, and chlamydiae were harvested by centrifugation of whole-culture lysates. For experiments requiring additional passages, total harvested chlamydiae were expanded onto fresh HeLa monolayers and cultivated for 24 hours in media containing PenG, aTc, and Theo. gDNA was harvested from ca. 10%–20% of pooled culture material using the Macherey-Nagel NucleoSpin Tissue kit according to the manufacturer’s instructions. WGS was accomplished via Illumina sequencing targeting ca. 10^6^ Mbp total reads (SeqCenter, LLC). Remaining material was used as source of mutant pools for isolation of individual mutants. Clonal strains were isolated from mutant output pools by limiting dilution in 384-well plates as described (54). Individual isolates were expanded, and WGS was accomplished by isolation of gDNA from pools containing equal IFU of five to six strains.

### Mapping and identification of specific mutants

Illumina WGS sequence reads were obtained from SeqCenter. Paired end reads and Phred scores of % > Q30 were obtained. All read alignments were completed using Geneious software (version 2023.2.1) with alignment to references from NCBI database. Read coverage was calculated as (read count × read length)/genome length. Assembled reads were aligned to the *C. trachomatis* LGV 434/Bu (NCBI accession no. NC_021049.1) genome sequence as described (22). Transposon insertions were determined by identifying the inverted repeat sequence in the mapped reads. Reads were also mapped to the transposon sequence to verify the insertion sites identified in the genome. For each insertion, an individual alignment file was generated with the CDS and the transposable element with inverted repeats in the annotated insertion site. A compiled table of reads was generated from insertion annotations, and the nomenclature for *C. trachomatis* D was used due to recognition in the field. A PCR screen was used to identify specific insertions within clonal isolates. Gene-specific reactions were performed on template DNA isolated from single strains withing a sequencing group using primers (Table S2) specific for the designated CDS and either the blaM-S or blaM-AS primers depending on the direction of insertions. The PCR products were resolved in 1% agarose gels that were imaged in the BioDoc-It Imager.

## Data Availability

All source data associated with this report have been deposited in publicly available databases. Sequence-independent data including raw data files of imaging data and spreadsheets containing analyzed data are available at Dryad (https://doi.org/10.5061/dryad.f1vhhmh6d). All sequence can be accessed at PRJNA1200737: A platform supporting generation and isolation of random transposon mutants in *Chlamydia trachomatis* (TaxID: 813). *Chlamydia trachomatis* strain:L2 (ID 1200737)—BioProject—NCBI.

## SUPPLEMENTAL MATERIAL

Supplemental tables jb.00500-24-s0001.docx Tables S1 to S3.

ASM does not own the copyrights to Supplemental Material that may be linked to, or accessed through, an article. The authors have granted ASM a non-exclusive, world-wide license to publish the Supplemental Material files. Please contact the corresponding author directly for reuse.
