# Supplementary material for: A platform supporting generation and isolation of random transposon mutants in Chlamydia trachomatis
Source: J Bacteriol. 2025 Feb 14;207(3):e00500-24. doi: 10.1128/jb.00500-24 (PMC11925237; doi:10.1128/jb.00500-24)
Supplement: Supplemental tables — Tables S1 to S3. [file jb.00500-24-s0001.docx]

**Table S1. Transposon insertions detected by WGS of pooled culture after random mutagenesis.**

| **Direction^a^** | **Gene^b^** | **Putative Function** | **Protein Change^c^** | **CTL^d^** | **CT^e^** |
| --- | --- | --- | --- | --- | --- |
| forward | intergenic | N/A | N/A | IG before CTL0201 | IG before CT829 |
| forward | *recC* | exodeoxyribonuclease V subunit gamma CDS | 673/1007aa | CTL0008 | CT640 |
| forward | *recC* | exodeoxyribonuclease V subunit gamma CDS | 104/1007aa | CTL0008 | CT640 |
| forward | intergenic | N/A | N/A | IG before CTL0247 | IG before CT868 |
| reverse | *dusB* | tRNA dihydrouridine synthase DusB | 319/335aa | CTL0011 | CT643 |
| reverse | *dusB* | tRNA dihydrouridine synthase DusB | 316/335aa | CTL0011 | CT643 |
| reverse | *dusB* | tRNA dihydrouridine synthase DusB | 247/335aa | CTL0011 | CT643 |
| forward | *dusB* | tRNA dihydrouridine synthase DusB | 119/335aa | CTL0011 | CT643 |
| reverse | intergenic | N/A | N/A | IG after CTL0348 | IG after CT093 |
| forward | Hypothetical CDS | DUF1347 family protein | 226/609aa | CTL0019 | CT651 |
| reverse | Hypothetical CDS | DUF1347 family protein | 272/609aa | CTL0643 | CT379 |
| forward | Hypothetical CDS | serine/threonine-protein kinase | 209/491aa | CTL0042 | CT673 |
| reverse | *parB* | ParB/RepB/Spo0J family partition protein | 146/282aa | CTL0057 | CT688 |
| forward | Hypothetical CDS | UvrB/UvrC motif-containing protein | 12/174aa | CTL0045 | CT676 |
| reverse | *parB* | ParB/RepB/Spo0J family partition protein | 245/282aa | CTL0057 | CT688 |
| reverse | intergenic | N/A | N/A | IG before CTL0059 | IG before CT690 |
| reverse | *tmeA* | type III secretion system effector TmeA | 297/323aa | CTL0063 | CT694 |
| forward | *tmeA* | type III secretion system effector TmeA | 211/399aa | CTL0063 | CT694 |
| forward | *tmeA* | type III secretion system effector TmeA | 308/399aa | CTL0063 | CT694 |
| forward | *secA* | preprotein translocase subunit SecA | 970 /970aa | CTL0070 | CT701 |
| reverse | hypothetical CDS | hypothetical protein | 67/176aa | CTL0071 | CT702 |
| forward | *pckA* | phosphoenolpyruvate carboxykinase (GTP) | 304/600aa | CTL0079 | CT710 |
| reverse | hypothetical CDS | MBL fold metallo-hydrolase | 79/263aa | CTL0107 | CT738 |
| reverse | intergenic | N/A | N/A | IG after CTL_t13 (tRNA-His) | N/A |
| forward | 23s rRNA | 23S ribosomal RNA | N/A | CTLr03 | N/A |
| forward | 23s rRNA | 23S ribosomal RNA | N/A | CTLr06 | N/A |
| forward | intergenic | N/A | N/A | IG before CTL0125 | IG before CT756 |
| reverse | intergenic | N/A | N/A | IG after CTL0125 | IG after CT756 |
| reverse | intergenic | N/A | N/A | IG before CTL0132 | IG before CT763 |
| forward | *mqnC* | cyclic dehypoxanthinyl futalosine synthase | 292/351aa | CTL0136 | CT767 |
| reverse | intergenic | N/A | N/A | IG before CTL0144 | IG before CT775 |
| forward | intergenic | N/A | N/A | IG before CTL0149 | IG before CT780 |
| reverse | uvrC | excinuclease ABC subunit UvrC | 135/599aa | CTL0159 | CT791 |
| reverse | uvrC | excinuclease ABC subunit UvrC | 29/599aa | CTL0159 | CT791 |
| reverse | Potential pseudogene | hypothetical protein | 1/89aa | CTL0161^f^ | CT793 |
| reverse | intergenic | N/A | N/A | N/A | N/A |
| forward | intergenic | N/A | N/A | IG before CTL0184 | IG before CT813 |
| forward | hypothetical CDS | hypothetical protein | 25/43aa | N/A | N/A |
| reverse | *tyrP* | aromatic amino acid transport family protein | 66/398aa | CTL0190 | CT818 |
| reverse | *tyrP* | aromatic amino acid transport family protein | 82/398aa | CTL0190 | CT818 |
| forward | *tyrP* | aromatic amino acid transport family protein | 384/398aa | CTL0190 | CT818 |
| reverse | intergenic | N/A | N/A | Ig between CTL0191 and CTL0192 | IG between CT819 and CT820 |
| reverse | *rmuC* | DNA recombination protein RmuC | 393/428aa | CTL0197 | CT825 |
| forward | *yggH* | tRNA (guanosine(46)-N7)-methyltransferase TrmB | 174/225aa | CTL0201 | CT829 |
| forward | *xerD* | site-specific tyrosine recombinase XerD | 301/ 301aa; | CTL0243 | CT864 |
| reverse | *glgB* | 1,4-alpha-glucan branching protein GlgB | 547/739aa | CTL0245 | CT866 |
| reverse | *dub2* | ChlaDub2 deubiquitinase | 329/340aa | CTL0246 | CT867 |
| reverse | *dub1* | ChlaDub1 deubiquitinase | 176/402aa | CTL0247 | CT868 |
| forward | *pmpF* | polymorphic outer membrane protein middle domain-containing protein | 366/966aa | CTL0249 | CT870 |
| reverse | intergenic | N/A | N/A | IG before CTL0251 | IG before CT872 |
| forward | hypothetical CDS | hypothetical protein | 93/116aa | N/A | N/A |
| forward | hypothetical CDS | DUF378 domain-containing protein | 13/91aa | CTL0256 | CT001 |
| forward | *cydA* | cytochrome ubiquinol oxidase subunit I | 249/447aa | CTL0268 | CT013 |
| reverse | hypothetical CDS | hypothetical protein | 219/243aa | CTL0271 | CT016 |
| reverse | *rnhB* | ribonuclease HII | 138/218aa | CTL0284 | CT029 |
| reverse | *glgX* | glycogen-debranching protein | 277/667aa | CTL0298 | CT042 |
| reverse | hypothetical CDS | hypothetical protein | 79/564aa | CTL0307 | CT051 |
| reverse | *hemW* | radical SAM family heme chaperone HemW | 105/377aa | CTL0308 | CT052 |
| forward | *hemW* | radical SAM family heme chaperone HemW | 11/377aa | CTL0308 | CT052 |
| forward | hemW; hypothetical protein CDS | radical SAM family heme chaperone HemW; hypothetical protein | hypothetical protein:149/149aa; hemW:3/377aa | hemW: CTL0308, hypothetical protein: CTL0309 | hemW: CT502, hypothetical protein: CT053 |
| reverse | *sucA* | 2-oxoglutarate dehydrogenase E1 component | 112/904aa | CTL0310 | CT054 |
| reverse | *sucA* | 2-oxoglutarate dehydrogenase E1 component | 626/904aa | CTL0310 | CT054 |
| reverse | *pgeF* | peptidoglycan editing factor PgeF | 196/244aa | CTL0312 | CT056 |
| forward | hypothetical CDS | membrane protein | 115/364aa | CTL0314 | CT058 |
| forward | hypothetical CDS | phosphatidylserine/phosphatidylglycerophosphate/cardiolipin synthase family protein | 241/362aa | CTL0339 | CT084 |
| forward | *ribF* | bifunctional riboflavin kinase/FAD synthetase | 264/302aa | CTL0348 | CT093 |
| forward | hypothetical CDS | hypothetical protein | 471/657aa | CTL0360 | CT105 |
| forward | *sfhB* | RluA family pseudouridine synthase | 82/304aa | CTL0361 | CT106 |
| forward | *sfhB* | RluA family pseudouridine synthase | 51/304aa | CTL0361 | CT106 |
| reverse | intergenic | N/A | N/A | IG before CTL0366 | IG before CT111 |
| forward | intergenic | N/A | N/A | IG before CTL0367 | IG before CT112 |
| forward | *incF* | inclusion membrane protein IncF | 65/105aa | CTL0372 | CT117 |
| reverse | *incG* | inclusion membrane protein IncG | 134/168aa | CTL0373 | CT118 |
| reverse | intergenic | N/A | N/A | IG between CTL0401 and CTL0402 | IG between CT146 and CT147 |
| reverse | hypothetical CDS | hypothetical protein | 39/1450aa | CTL0402 | CT147 |
| reverse | intergenic | N/A | N/A | IG before CTL0418 | N/A |
| reverse | hypothetical CDS | hypothetical protein | 69/87aa | CTL0419A | N/A |
| reverse | hypothetical CDS | hypothetical protein | 63/164aa | CTL0425 | N/A |
| forward | *zwf* | glucose-6-phosphate dehydrogenase | 352/508aa | CTL0437 | CT185 |
| reverse | hypothetical CDS | hypothetical protein | 207/364aa | CTL0447 | CT195 |
| reverse | hypothetical CDS | hypothetical protein | 166/364aa | CTL0447 | CT195 |
| forward | hypothetical CDS | hypothetical protein | 35/364aa | CTL0447 | CT195 |
| reverse | hypothetical CDS | hypothetical protein | 81/107aa | CTL0448 | CT196 |
| reverse | *hemL* | glutamate-1-semialdehyde 2,1-aminomutase | 183/423aa | CTL0462 | CT210 |
| reverse | hypothetical CDS | hypothetical protein | 69/148aa | CTL0477 | CT224 |
| reverse | hypothetical CDS | hypthetical protein | 86/166aa | CTL0478 | CT226 |
| forward | *yidC* | membrane protein insertase YidC | 636/788aa | CTL0503 | CT251 |
| reverse | intergenic | N/A | N/A | IG before CTL0512 | IG before CT260 |
| forward | hypothetical CDS | hypothetical protein | 2/96aa | CTL0523 | CT271 |
| reverse | hypothetical CDS | hypothetical protein | 159/220aa | CTL0529 | CT277 |
| reverse | *incM* | Inclusion membrane protein | 561/565aa | CTL0540 | CT288 |
| forward | intergenic | N/A | N/A | IG between CTL0541 and CTL0542 | IG between CT289 and CT290 |
| forward | *mrsA* | phospho-sugar mutase | 128/594 | CTL0547 | CT295 |
| forward | intergenic | N/A | N/A | IG before CTL0576 | IG before CT324 |
| reverse | *xseA* | exodeoxyribonuclease VII large subunit | 471/517aa | CTL0583 | CT329 |
| forward | *uvrA* | excinuclease ABC subunit UvrA | 109/1787aa | CTL0587 | CT333 |
| forward | intergenic | N/A | N/A | IG after CTL0597 | IG after CT343 |
| forward | hypothetical CDS | hypothetical protein | 45/122 aa | CTL0599 | CT345 |
| forward | *rsmA* | 16S rRNA (adenine(1518)-N(6)/adenine(1519)-N(6))- dimethyltransferase RsmA | 278/278aa | CTL0608 | CT354 |
| forward | Potential pseudogene | hypothetical protein | 143/178aa | CTL0612^f^ | CT358 |
| forward | BioY | biotin transporter BioY | 100/197aa | CTL0613 | CT359 |
| reverse | hypothetical CDS | DUF2608 domain-containing protein | 92/262aa | CTL0625 | CT371 |
| reverse | hypothetical CDS | DUF2608 domain-containing protein | 225/ 262aa | CTL0625 | CT371 |
| forward | hypothetical CDS | DUF2608 domain-containing protein | 260/262aa | CTL0625 | CT371 |
| reverse | *pgi* | glucose-6-phosphate isomerase | 318/526aa | CTL0633 | CT378 |
| reverse | *hflX* | GTPase HflX | 428/448aa | CTL0643 | CT379 |
| forward | *hflX* | GTPase HflX | 383/448aa | CTL0643 | CT379 |
| reverse | *phnP* | MBL fold metallo-hydrolase | 83/267 | CTL0635 | CT380 |
| forward | *phnP* | MBL fold metallo-hydrolase | 36/267aa | CTL0635 | CT380 |
| forward | intergenic | N/A | N/A | IG before CTL0725 | IG before CT465 |
| reverse | *pmpA* | Pmp family polymorphic membrane protein autotransporter adhesin | 214/976aa | CTL0669 | CT412 |
| reverse | *pmpA* | Pmp family polymorphic membrane protein autotransporter adhesin | 700/976aa | CTL0669 | CT412 |
| reverse | *pmpA* | Pmp family polymorphic membrane protein autotransporter adhesin | 976/976aa | CTL0669 | CT412 |
| forward | *pmpB* | polymorphic outer membrane protein middle domain-containing protein | 260/1750aa | CTL0670 | CT413 |
| forward | *pmpB* | polymorphic outer membrane protein middle domain-containing protein | 674/1750aa | CTL0670 | CT413 |
| reverse | *pmpB* | polymorphic outer membrane protein middle domain-containing protein | 984/1750aa | CTL0670 | CT413 |
| forward | *pmpB* | polymorphic outer membrane protein middle domain-containing protein | 1088/1750 | CTL0670 | CT413 |
| forward | *pmpB* | polymorphic outer membrane protein middle domain-containing protein | 1211/1750aa | CTL0670 | CT413 |
| reverse | *pmpB* | polymorphic outer membrane protein middle domain-containing protein | 1580/1750 | CTL0670 | CT413 |
| reverse | Hypothetical CDS | small basic protein | 53/54 | CTL0680 | CT412.2 |
| reverse | intergenic | N/A | N/A | IG after CTL0683 | IG after CT424 |
| forward | *truA* | HAD family phosphatase | 104/225aa | CTL0724 | CT464 |
| reverse | intergenic | N/A | N/A | IG after CTL0725 | IG after CT465 |
| reverse | *recO* | DNA repair protein RecO | 20/244aa | CTL0730 | CT470 |
| reverse | Hypothetical CDS | methylated-DNA--[protein]-cysteine S-methyltransferase | 27/171aa | CTL0738 | CT477 |
| reverse | Hypothetical CDS | methylated-DNA--[protein]-cysteine S-methyltransferase | 160/171aa | CTL0738 | CT477 |
| forward | intergenic | N/A | N/A | IG after CTL0742 | IG after CT481 |
| forward | hypothetical CDS | hypothetical protein | 122/218aa | CTL0743 | CT482 |
| reverse | hypothetical CDS | hypothetical protein | 31/218aa | CTL0743 | CT482 |
| reverse | *vldD* | acyl-CoA thioesterase | 24/161aa | CTL0797 | CT535 |
| reverse | *uhpC* | MFS transporter | 454/457aa | CTL0806 | CT544 |
| forward | *fmu* | RsmB/NOP family class I SAM-dependent RNA methyltransferase | 101/326aa | CTL0815 | CT553 |
| reverse | intergenic | N/A | N/A | IG after CTL0817 | IG after CT554 |
| reverse | intergenic | N/A | N/A | IG between CTL0827 and CTL0828 | IG between CT564 and CT565 |
| reverse | intergenic | N/A | N/A | IG before CTL0843 | IG before CT580 |
| forward | intergenic | N/A | N/A | IG before CTL0845 | IG before CT582 |
| reverse | *sdhB* | succinate dehydrogenase iron-sulfur subunit | 173/256aa | CTL0854 | CT591 |
| reverse | Hypothetical CDS | TenA family transcriptional regulator | 119/232aa | CTL0874 | CT610 |
| forward | Hypothetical CDS | TenA family transcriptional regulator | 106/232aa | CTL0874 | CT610 |
| reverse | Hypothetical CDS | rhodanese-related sulfurtransferase | 12/328aa | CTL0891 | CT627 |
| reverse | *pgp7* | Plasmid-encoded Integrase | 160/305aa | NA | pCT07 |
| Reverse | *pgp7* | Plasmid-encoded Integrase | 20/305aa | NA | pCT07 |
| reverse | *pgp3* | Plasmid-encoded secreted protein | 104/265 aa | NA | pCT03 |
|  |  |  |  |  |  |

^a^ Direction indicates orientation of the transposable to the sense (forward) or anti-sense (reverse) strand of the chromosome.

^b^ Gene designations are listed as indicated in annotated chlamydial genome (1).

^c^ Protein change is indicated by the last residue before transposon direction/total number of residues in the CDS.

^d^ Genes designations are derived from the L2 Chromosome annotation (2).

e Corresponding gene designation form the *C. trachomatis* serovar D genome is indicated base on (1).

^f^  Genes are listed as potential pseudogenes in the L2 genome (2).

| **Target** | **Primer name** | **Primer Sequence (5’→ 3’)** |
| --- | --- | --- |
| **Screening Primers** | | |
| *Chlamydia-specific* |  | |
|  | CT006-F1 | CTTAAAATCGAAATAGCGGC |
|  | CT037-F1 | TGGAGTTCAAGATAGTCCC |
|  | CT048-F1 | GTTGGAGAGATTGTTCGG |
|  | CT193-F2 | TGAGCCAAATGCGGGTATCTGGG |
|  | CT290-F1 | GTTCGAGTTGGATCCGG |
|  | CT406-R2 | GATCCTTGTTTACTACGATAGGCCCTTC |
|  | CT412-F1 | GAAACTCCCTTCTTCTCACCTAAGGG |
|  | CT625-F1 | ACATTAACTCAAGAAATGGTGG |
|  | CT627-F1 | TCTGAAGAAGGGATTAACGG |
|  | CT829-F3 | ATAATCTGCTTATTGTCTGTGG |
|  |  |  |
| *blaM* |  |  |
|  | blaM-F1 | ATCATTGGAAAACGTTCTTCGGG |
|  | blaM-AS | CAGTGAGGCACCTATCTCAGCGATCTG |
|  | blaM-R | CTGGATCTCAACAGCGGTAAGATCC |

**Supplementary Table 2. Custom Primers**

**Supplementary Table 3. Expected PCR product size from clonal isolates.**

| Insertion Target^a^ | Screening Primer 1 | Screening Primer 2 | Predicted Amplicon (bp)^b^ |
| --- | --- | --- | --- |
| IG between *ct_005* (*ctl0260*) & *ct_006* (*ctl0261*) | CT006-F1 | blaM-F1 | 613 |
| *ct_037* | CT037-F1 | blaM-AS | 307 |
| *ct_048* (*ctl0304*) | CT048-F1 | blaM-AS | 425 |
| *ct_192* (*ctl0444*) | CT193-F2 | blaM-F1 | 700 |
| IG between *ct_289* (*ctl0541*) & *ct_290* (*ctl0542*) | CT290-F1 | blaM-AS | 1434 |
| IG upstream of *ct_406* (*ctl0663*) | CT406-R2 | blaM-R | 1000 |
| *ct_412* (*ctl0669*) | CT412-F1 | blaM-AS | 938 |
| *ct_625* (*ctl0889*) | CT625-F1 | blaM-AS | 880 |
| *ct_627* (*ctl0891*) | CT627-F1 | blaM-AS | 842 |
| *ct_829* (*ctl0201*) | CT829-F3 | blaM-F1 | 628 |

^a^ Insertion target reflects the corresponding genomic locus predicted to harbor the transposon insertion

^b^ Amplicon size is predicted from insertion mapping of transposon position and reflects the PCR product produced with a transposon-specific and *Chlamydia*-specific primer pair.

**REFERENCES**

1. **Stephens RS, Kalman S, Lammel C, Fan J, Marathe R, Aravind L, Mitchell W, Olinger L, Tatusov RL, Zhao Q, Koonin EV, Davis RW.** 1998. Genome sequence of an obligate intracellular pathogen of humans: *Chlamydia trachomatis*. Science **282:**754-759.

2. **Thomson NR, Holden MT, Carder C, Lennard N, Lockey SJ, Marsh P, Skipp P, O'Connor CD, Goodhead I, Norbertzcak H, Harris B, Ormond D, Rance R, Quail MA, Parkhill J, Stephens RS, Clarke IN.** 2008. *Chlamydia trachomatis*: genome sequence analysis of lymphogranuloma venereum isolates. Genome Res **18:**161-171.
